# Blood Cell Mitochondrial Respiration Increases With Age and Varies by Sex in Healthy Adults

**DOI:** 10.1111/acel.70387

**Published:** 2026-01-21

**Authors:** Howard J. Phang, Jaclyn Bergstrom, Benjamin Keri, Stephanie R. Heimler, Stephen Dozier, Lina M. Scandalis, David Wing, Daniel Moreno, Nina N. Sun, Anthony J. A. Molina

**Affiliations:** ^1^ School of Medicine University of California San Diego La Jolla California USA; ^2^ Skaggs School of Pharmacy and Pharmaceutical Sciences University of California San Diego La Jolla California USA; ^3^ University of Puget Sound Tacoma Washington USA; ^4^ Herbert Wertheim School of Public Health and Human Longevity Science University of California San Diego La Jolla California USA; ^5^ Sam and Rose Stein Institute for Research on Aging University of California, San Diego La Jolla California USA

## Abstract

Mitochondrial dysfunction is recognized as a biological hallmark of aging; however, bioenergetic capacity across the healthy human life course remains insufficiently characterized. While aging is generally associated with a systemic decline in mitochondrial function (“age‐related bioenergetic decline”), recent research suggests that age‐related bioenergetic differences are context dependent. Blood cells are extensively utilized as accessible samples for human bioenergetic profiling; therefore, our goal was to characterize bioenergetic capacity in platelets, peripheral blood mononuclear cells (PBMCs), monocytes, and lymphocytes of healthy adults from the San Diego Nathan Shock Center Clinical Cohort representative of the adult life course (20–80+ years of age). In our sample of 72 adults, we found that chronological age was positively associated with PBMC (maximal respiration [Max] *β* = 0.147, *p* = 0.028) and lymphocyte respiratory capacity (Max *β* = 0.135, *p* = 0.041). Notably, the pattern of age‐related differences varied by sex; age showed a weak positive association with platelet respiration (Max *β* = 0.219, *p* = 0.037) in men but not in women. Similarly, age showed a strong positive association with PBMC respiration (Max *β* = 0.206, *p* = 0.018) in women but not in men. We also explored the relationship between glycolysis and respiration and found strong positive associations in platelets, PBMCs, and monocytes, but not lymphocytes. It is possible that, despite our cohort consisting of healthy, disease‐free individuals, the elevated respiratory capacity in older adults may be reflective of compensatory mechanisms that require further investigation. Nonetheless, these findings underscore the importance of considering biological context, such as donor health, sex, and tissue type, in understanding age‐related bioenergetic differences.

## Graphical abstract

Using samples from the San Diego Nathan Shock Center Clinical Cohort, we found that blood cell bioenergetic capacity is generally positively associated with age and sex dependent. These findings highlight the importance of biological context in bioenergetics research. Created in BioRender. Lab, M. (2025) https://BioRender.com/pvgt48f.

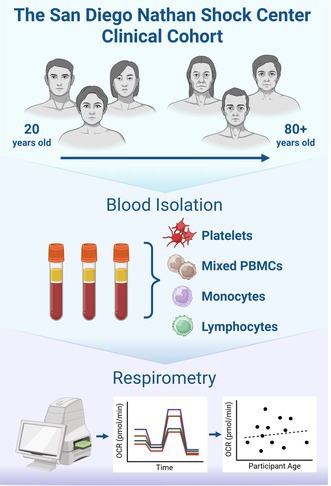

## 1 Introduction

It is well established that detrimental changes in mitochondrial function over time are associated with aging, evidenced by a multitude of animal and human studies (Lenaz et al. 2000; Bratic and Larsson 2013; Sun et al. 2016). This has supported the recognition of mitochondrial dysfunction as a biological hallmark of aging (López‐Otín et al. 2023). Further, mitochondrial dysfunction appears to play a significant role in the development of age‐related phenotypes, ranging from normal physiology like physical and cognitive function to pathological conditions like sarcopenia and neurodegenerative disorders (Bowling and Beal 1995; Short et al. 2005). This suggests that mitochondrial function may be a useful biomarker to evaluate age‐related health and decline.

In order to advance its utility as a clinical biomarker, there is a requisite need to understand how age‐related bioenergetic function changes across the human life course. While bioenergetic decline is generally accepted as a hallmark of aging, it is less clearly defined how mitochondrial health and function should be assessed. Much of the early research on age‐related bioenergetic decline has focused on skeletal muscle, which has provided valuable foundational insights on respiratory capacity and adenosine triphosphate (ATP) production (Shigenaga et al. 1994; Rooyackers et al. 1996). However, recent studies suggest that skeletal muscle bioenergetic capacity may be more closely linked to physical activity levels and fitness than to chronological age alone (Lee et al. 2024; Distefano et al. 2017). This raises questions about the generalizability of findings from skeletal muscle to other tissues and systems. Recent research also suggests that age‐related differences in mitochondrial function are influenced by the donor organ, tissue, and sex. For example, Jedlička et al. (2022) demonstrated in rats that skeletal muscle respiration decreased with age while brain tissue respiration remained stable and platelet respiration increased. These findings underscore the complexity of biological aging and suggest that a uniform concept of “age‐related bioenergetic decline” may be overly simplistic or even misleading. It is also important to recognize that higher respiration as opposed to lower does not necessarily equate to better mitochondrial function, without considering the contributions of other factors like cell function and type. Thus, the meaning of “age‐related bioenergetic decline” must be reexamined and refined to reflect the specific biological contexts in which these changes occur, ensuring a more accurate and comprehensive understanding of mitochondrial aging across different tissues and systems.

Blood‐based bioenergetic profiling is a popular, minimally invasive tool to investigate human mitochondrial function and its relationship to aging. Importantly, blood cell respiration has been correlated with various other age‐related markers of health, including physical function, gait speed, and resting metabolic rate (Tyrrell et al. 2015; Heimler et al. 2022). Since blood provides a highly accessible and reliable source of biological material, it is important to determine how blood cells reflect and report on age‐related bioenergetic decline.

Our goal was to characterize age‐related differences in the bioenergetic capacity of blood cells in healthy adults from the San Diego Nathan Shock Center (SD‐NSC) Clinical Cohort. Although most blood‐based bioenergetics studies focus solely on platelets and mixed peripheral blood mononuclear cells (PBMCs), we further isolated PBMCs into their constituent cell types, monocytes and lymphocytes, to achieve greater cell‐specific insights. We hypothesized that bioenergetic capacity, including respiratory and glycolytic capacity, of platelets, mixed PBMCs, isolated monocytes, and isolated lymphocytes is associated with chronological age of the donor. We also explored the relationship between glycolytic capacity and respiratory capacity in these cells.

## 2 Methods

### 2.1 Study Design

The SD‐NSC Clinical Cohort (IRB #201141) was established in 2021 to provide a resource of biospecimens and extensive phenotypic data from adult participants across the human adult life course to support studies of aging. It comprises 75 healthy, community‐dwelling adults who are free from chronic conditions and selected through strict inclusion and exclusion criteria. The selection process aimed to minimize biological variability and maintain a focus on non‐pathological aging. The cohort design, recruitment, and inclusion processes have been described previously (Phang et al. 2024). To summarize, participants were excluded if they were pregnant, had diabetes, uncontrolled hypertension, any other heart or cardiovascular condition (coronary artery disease, congestive heart failure, atrial fibrillation, etc.), cancer or history of cancer within 5 years, dementia or other cognitive impairments, sensory or physical impairments, neurological conditions, or active respiratory disease. We also excluded individuals who were actively taking supplements that may interfere with our measurements or biological outcomes, including but not limited to NAD+ supplements, MitoQ, or other mito‐modulatory medications. For the present study, we selected participants based on available lymphocyte outcome data, which constitute the highest proportion of mixed PBMCs. We included 72 participants with reported lymphocyte maximal respiration.

### 2.2 Sample Collection

We centrifuged three Acid‐Citrate Dextrose‐Anticoagulant (ACD‐A) tubes containing 8 mL of fresh whole blood at 500 × *g* for 15 min to separate platelet‐rich plasma, buffy coat, and red blood cells. After separation of platelet‐rich plasma from the buffy coat and red blood cells, each cell isolation was done independently and in parallel. While there are many cell types present in whole blood (e.g., 1%–2% dendritic cells, 0.1%–0.2% stem cells, etc.), our isolation methods focus on platelets, mixed PBMCs, monocytes, and lymphocytes.

### 2.3 Platelet Isolation

We collected and pooled platelet‐rich plasma with added Prostaglandin E1 (PGE1) (Cayman, CAY13010) in excess (~1 M stock, 200 nM working concentration) to prevent platelet activation, then centrifuged the tube at 1500 × *g* for 10 min. Platelet‐free plasma was removed and further centrifuged at 2000 × *g* for 10 min before storage at −80°C. The remaining platelet pellet was resuspended in 4 mL of Dulbecco's Phosphate Buffered Saline (DPBS, Gibco, 14190144), with PGE1 added, and centrifuged at 1500 × *g* for 7 min. After aspirating the supernatant, the pellet was resuspended in 1 mL of DPBS with PGE1 added for a final cell count using a Beckman Coulter DxH 520 Hematology Analyzer (Brea, CA).

### 2.4 Peripheral Blood Mononuclear Cell (PBMC) Isolation

We collected and pooled the buffy coat with added PGE1 to prevent aggregation of any errant platelets, then diluted the sample to 25 mL with Roswell Park Memorial Institute 1640 (RPMI) media (Gibco, 11875093). This solution was taken and carefully layered on top of Histopaque 1077 (Sigma, 10771), then centrifuged at 700 × *g* for 30 min. After spinning, the buffy coat floating atop the histopaque layer was collected, pooled together, and further diluted to approximately 25 mL with RPMI media. This solution was centrifuged at 700 × *g* for 15 min. After aspirating the supernatant, the pellet was resuspended in 1 mL of RPMI with PGE1 added and centrifuged at 400 × *g* for 5 min. After aspirating the supernatant, the pellet was resuspended in 1 mL of RPMI with PGE1 added for a final cell count.

### 2.5 Monocyte Isolation

After reserving an aliquot of mixed PBMCs for experimentation, we centrifuged the remaining PBMCs at 500 × *g* for 5 min. After aspirating the supernatant, the cells are resuspended in 120 μL of RPMI + bovine serum albumin (BSA) buffer and 30 μL of CD14+ MACS microbeads (Miltenyi Biotec, 130‐050‐201), which selectively bind to monocytes. This solution was incubated on a tube rocker at room temperature for 15 min. After incubation, 500 μL of RPMI + BSA buffer was added, and the resulting solution is centrifuged at 500 × *g* for 4 min. After aspirating the supernatant, the pellet was resuspended in 1 mL of RPMI + BSA buffer. This solution was run through a MACS LS Column (Miltenyi Biotec) and washed with 5 mL of RPMI + BSA buffer. The total 6 mL of CD14‐ solution was collected for lymphocyte isolation. The column was removed from the magnetic stand, and 6 mL of RPMI + BSA buffer was used to plunge out CD14+ cells from the column. The CD14+ sample was centrifuged at 500 × *g* for 4 min. After aspirating the supernatant, the pellet was resuspended in 1 mL of RPMI for a final cell count.

### 2.6 Lymphocyte Isolation

We centrifuged the CD14‐ solution at 500 × *g* for 4 min. After aspirating the supernatant, the pellet was resuspended in 120 μL of RPMI + bovine serum albumin (BSA) buffer and 30 μL of CD61+ MACS microbeads (Miltenyi Biotec, 130‐051‐101), which selectively bind to platelets. This solution was incubated on a tube rocker at room temperature for 15 min. After incubation, 500 μL of RPMI + BSA buffer was added and the resulting solution was centrifuged at 500 × *g* for 4 min. After aspirating the supernatant, the pellet was resuspended in 1 mL of RPMI + BSA buffer. This solution was run through a MACS LS Column (Miltenyi Biotec) and washed with 5 mL of RPMI + BSA buffer. The total 6 mL of CD61‐solution was centrifuged at 500 × *g* for 4 min, and CD61+ cells were disposed of (errant platelets). After aspirating the supernatant, the pellet was resuspended in 1 mL of RPMI for a final cell count.

### 2.7 Respirometry

After obtaining a final count of each cell type, we plated cells on an XFe96‐well plate for respirometric analysis on the Agilent Seahorse XFe96 (Billerica, MA). We plated the following densities per cell type: 20 M/well for platelets, 0.3 M/well for PBMCs, 0.3 M/well for monocytes, and 0.5 M/well for lymphocytes, with at least 4 technical replicates per cell type. These cell densities were optimized to provide the most robust Basal and Max values. Once the cells were plated, we spun the plate at 200 × *g* for 2 min to ensure cells were attached to the plate.

We utilized the Mito Stress Test assay which allows measurement of our primary bioenergetic outcomes. This protocol measures oxygen consumption rate (OCR) to provide a dynamic measurement that is sensitive and responsive to various perturbations. Addition of 1.25 μM oligomycin (ATP synthase inhibitor), 1 μM carbonyl cyanide‐p‐trifluoromethoxyphenylhydrazone (FCCP, uncoupler), 1 μM rotenone (complex I inhibitor), and 1 μM antimycin A (complex III inhibitor) allows us to capture the bioenergetic parameters basal respiration (Basal) and maximal uncoupled respiration (Max). Spare respiratory capacity (SRC) was calculated as the difference between Max and Basal. Detailed methodologies can be found in our previous publication.

### 2.8 Measurement of Glycolysis

The Agilent Seahorse XFe96 allows concurrent measurement of glycolytic flux in the form of proton efflux rate, which has been shown to be directly correlated to other measures of glycolytic function such as cellular lactate efflux (Romero et al. 2021). To calculate glycolytic function, we used the glycolytic proton efflux rate (GlycoPER) calculation as described previously (Romero et al. 2021). Briefly, to convert extracellular acidification rate (ECAR) to GlycoPER, several experimental constants must be determined to transform the data. Multiplying ECAR by the buffer factor, volume scaling factor, and volume of the measurement chamber yields proton efflux rate (PER). This PER must then be corrected for non‐glycolytic media acidification in the form of CO_2_ emitted from oxidative phosphorylation, which we calculated by multiplying OCR by the CO_2_ contribution factor. The resulting value is GlycoPER, which represents the proton efflux that constitutes glycolytic activity. We used ECAR or GlycoPER values following oligomycin injection to capture stimulated maximal glycolytic flux.

### 2.9 Covariates

We measured covariates as previously described (Phang et al. 2024). Briefly, we measured BMI using well‐established formulas (kg/m^2^). We captured physical activity using the ActiGraph GT3X + accelerometer for wireless accelerometry, utilizing average moderate‐to‐vigorous physical activity (MVPA) per day as our measure of overall physical activity.

### 2.10 Statistical Analyses

We used R version 4.2.2 to conduct all statistical analyses. Participant characteristics were summarized using descriptive statistics, and sex and age‐group differences were tested using *t*‐tests and chi‐squared tests as appropriate. Bioenergetic parameters were sex‐specifically standardized for use in further analysis. Associations between bioenergetic parameters and age were tested using linear regression in models that were unadjusted, as well as models adjusted for BMI. All respiration and GlycoPER values were normalized by cell number.

## 3 Results

### 3.1 Participant Characteristics

Participant characteristics are reported in Table 1 stratified by age groups of 20 years. Of the 72 participants, ages ranged from 23 to 82 years of age, and 57% were women. Based on previous literature reporting differences in bioenergetic capacity based on sex, we conducted analysis both in aggregate and sex‐stratified (Mahapatra et al. 2024). Sex‐stratified participant characteristics are reported in Table S1. Blood cell composition remained generally consistent across age groups, except for the absolute count of monocytes (*p* = 0.037) and lymphocytes (*p* = 0.009). Regardless, their percent abundance in PBMCs was consistent with age (*p* = 0.233 and *p* = 0.439 respectively).

**TABLE 1:** Participant characteristics.

	*N*	All	20–40	40–60	60–80+	*p*
*N*		72	20	20	32	
Female, *N* (%)	72	41 (57%)	10 (50%)	13 (65%)	18 (56%)	0.628
Body max index (BMI), mean (SD)	72	24.2 (2.9)	24.94 (3.00)	24.73 (2.66)	23.45 (2.90)	0.129
Platelet count (million/mL), mean (SD)	72	189.20 (45.75)	192.05 (59.00)	183.98 (39.39)	190.68 (41.04)	0.834
PBMC count (million/mL), mean (SD)	72	1.62 (0.49)	1.91 (0.57)	1.48 (0.27)	1.53 (0.48)	0.006
Monocyte count (million/mL), mean (SD)	71	0.33 (0.09)	0.37 (0.11)	0.30 (0.05)	0.33 (0.09)	***0.037***
Lymphocyte count (million/mL), mean (SD)	72	1.29 (0.43)	1.54 (0.50)	1.19 (0.26)	1.21 (0.42)	***0.009***
Monocyte % of PBMCs, mean (SD)	70	23.95 (4.63)	22.90 (4.85)	23.36 (3.63)	24.97 (4.95)	0.233
Lymphocyte % of PBMCs, mean (SD)	72	74.79 (5.04)	75.41 (5.87)	75.55 (3.63)	73.94 (5.25)	0.439

Abbreviations: PBMC, peripheral blood mononuclear cells; SD, standard deviation.

*Note*: Bold values represent statistical significance (*p* < 0.05).

### 3.2 Bioenergetic Capacity of PBMCs and Lymphocytes Are Associated With Age

**FIGURE 1:**
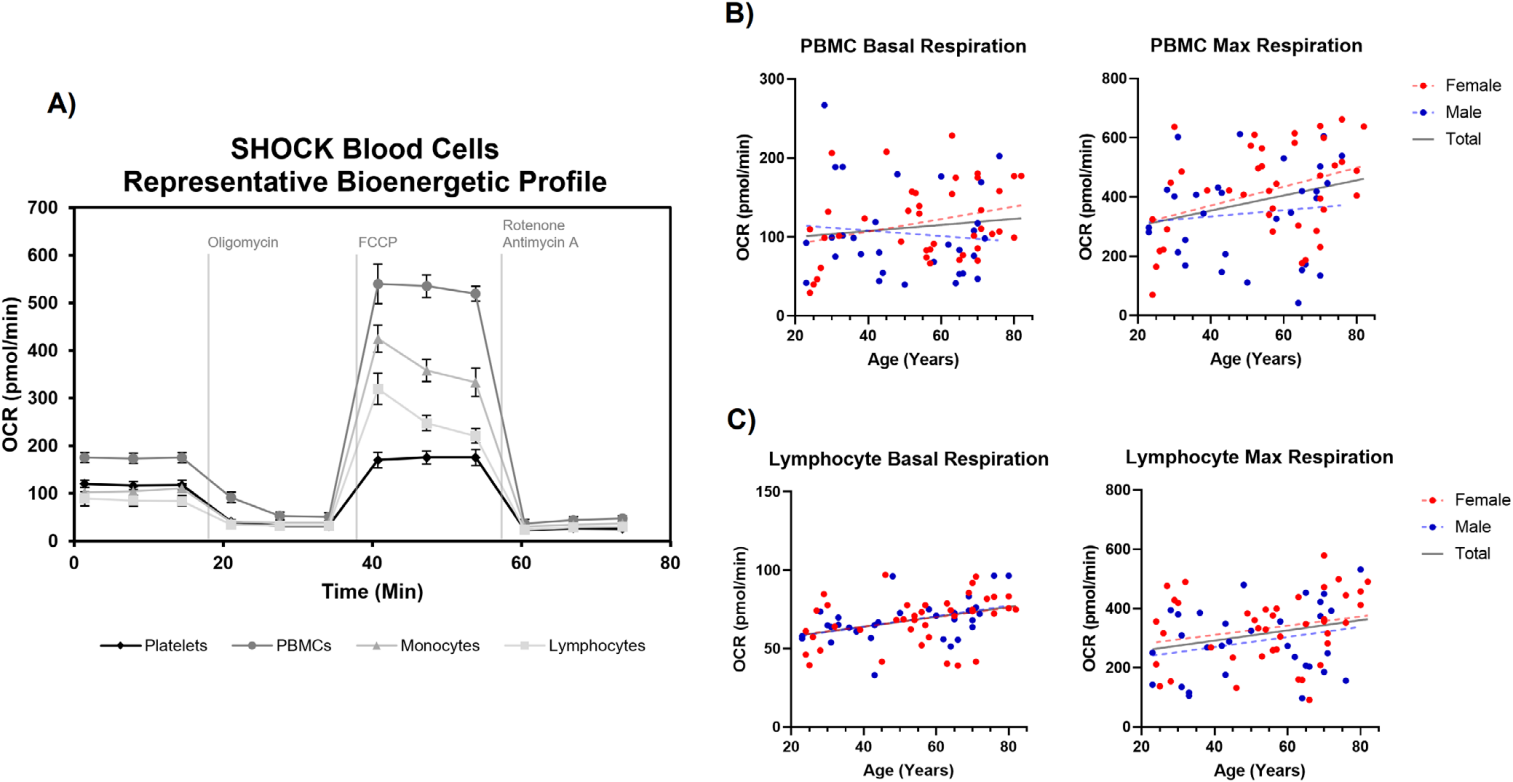
Representative scatterplots of the association between age and blood cell bioenergetic capacity. (A) Representative bioenergetic profile of one SD‐NSC participant including platelets, PBMCs, monocytes, and lymphocytes. Unadjusted (M0) Basal and Max associated with age in (B) PBMCs and (C) lymphocytes. Red points represent women and blue represent men. OCR, oxygen consumption rate; PBMC, peripheral blood mononuclear cells.

First, we examined how chronological age related to bioenergetic parameters Basal, Max, SRC, and GlycoPER in platelets, mixed PBMCs, monocytes, and lymphocytes (Table 2). In unadjusted models (M0), we found positive associations between age and PBMC Max (*β* = 0.147, *p* = 0.028) and PBMC SRC (*β* = 0.158, *p* = 0.018). Adjustments for BMI (M1) did not significantly affect these relationships. We also found positive associations between age and lymphocyte Basal (*β* = 0.216, *p* = 0.001) and lymphocyte Max (*β* = 0.135, *p* = 0.041). Adjustments for BMI (M1) strengthened these associations, particularly for SRC (*β* = 0.140, *p* = 0.040). We did not identify any associations between age and bioenergetic parameters in platelets or monocytes. Representative graphs and scatterplots are shown in Figure 1. Sensitivity analysis in a subset of participants with available physical activity measurements indicated that physical activity had little impact on the magnitude and/or directionality of these relationships (Table S2).

**TABLE 2:** Standardized regression parameters of age for blood cell bioenergetic parameters.

		Basal		Max		SRC		GlycoPER	
		*β*	*p*	*β*	*p*	*β*	*p*	*β*	*p*
Platelet (*n* = 72)	M0	0.061	0.372	0.091	0.178	0.073	0.283	0.121	0.073
	M1	0.045	0.521	0.083	0.238	0.065	0.353	0.091	0.179
PBMCs (*n* = 72)	M0	0.072	0.288	***0.147***	***0.028***	***0.158***	***0.018***	0.122	0.067
	M1	0.041	0.552	0.126	0.068	***0.143***	***0.039***	0.099	0.145
Monocyte (*n* = 71)	M0	0.027	0.684	−0.011	0.872	−0.016	0.807	0.064	0.340
	M1	0.042	0.542	0.012	0.863	0.006	0.935	0.082	0.242
Lymphocyte (*n* = 72)	M0	***0.216***	***0.001***	***0.135***	***0.041***	0.116	0.081	0.083	0.221
	M1	***0.237***	***< 0.001***	***0.161***	***0.018***	***0.140***	***0.040***	0.099	0.152

Abbreviations: GlycoPER, glycolytic proton efflux rate; M0, unadjusted; M1, adjusted for BMI; PBMC, peripheral blood mononuclear cells; SRC, spare respiratory capacity.

*Note*: Bold values represent statistical significance (*p* < 0.05).

### 3.3 Relationships Between Bioenergetic Capacity and Age Are Different Between Men and Women

We examined how these associations differ when stratified by participant sex (Table 3). In platelets, we found a positive association between age and Max in men (*β* = 0.219, *p* = 0.037) and similar positive trends across all other bioenergetic parameters. In contrast, we found no associations between age and platelet bioenergetic parameters in women. In PBMCs, we found positive associations between age and Max (*β* = 0.206, *p* = 0.018), SRC (*β* = 0.193, *p* = 0.028), and GlycoPER (*β* = 0.217, *p* = 0.012) in women, but not in men. We found no associations between age and monocyte bioenergetic parameters in men nor women. In lymphocytes, we found positive correlations between age and basal respiration in men (*β* = 0.250, *p* = 0.013) and women (*β* = 0.195, *p* = 0.025), with similar positive trends across all other bioenergetic parameters. Adjustments for BMI (M1) did not significantly affect any of the associations. Sensitivity analysis in the subset of men and women with available physical activity measurements indicated that physical activity had little impact on the magnitude and/or directionality of these relationships (Table S3).

**TABLE 3:** Standardized regression parameters of age for blood cell bioenergetic parameters, stratified by sex.

		Basal		Max		SRC		GlycoPER	
		*β*	*p*	*β*	*p*	*β*	*p*	*β*	*p*
Men									
Platelet (*n* = 31)	M0	0.189	0.076	***0.219***	***0.037***	0.177	0.097	0.201	0.063
	M1	0.200	0.064	***0.232***	***0.029***	0.187	0.084	0.198	0.072
PBMC (*n* = 31)	M0	−0.060	0.587	0.066	0.550	0.112	0.311	−0.008	0.943
	M1	−0.068	0.555	0.047	0.678	0.090	0.416	−0.010	0.928
Monocyte (*n* = 30)	M0	−0.106	0.312	−0.043	0.687	−0.025	0.812	−0.020	0.854
	M1	−0.098	0.361	−0.041	0.709	−0.025	0.818	−0.007	0.951
Lymphocyte (*n* = 31)	M0	***0.250***	***0.013***	0.142	0.173	0.121	0.249	0.029	0.791
	M1	***0.259***	***0.012***	0.154	0.148	0.132	0.217	0.035	0.749
Women									
Platelet (*n* = 41)	M0	−0.028	0.755	0.004	0.966	0.002	0.985	0.070	0.434
	M1	−0.094	0.312	−0.046	0.625	−0.040	0.672	0.004	0.962
PBMC (*n* = 41)	M0	0.163	0.065	***0.206***	***0.018***	***0.193***	***0.028***	***0.217***	***0.012***
	M1	0.115	0.200	***0.190***	***0.039***	***0.192***	***0.039***	***0.176***	***0.048***
Monocyte (*n* = 41)	M0	0.126	0.158	0.012	0.891	−0.010	0.910	0.124	0.163
	M1	0.151	0.111	0.061	0.511	0.039	0.672	0.136	0.153
Lymphocyte (*n* = 41)	M0	***0.195***	***0.025***	0.132	0.138	0.114	0.202	0.123	0.171
	M1	***0.222***	***0.017***	0.168	0.074	0.148	0.115	0.145	0.127

Abbreviations: GlycoPER, glycolytic proton efflux rate; M0, unadjusted; M1, adjusted for BMI; PBMC, peripheral blood mononuclear cells; SRC, spare respiratory capacity.

*Note*: Bold values represent statistical significance (*p* < 0.05).

### 3.4 Glycolytic Function Is Positively Associated With Respiratory Capacity

**FIGURE 2:**
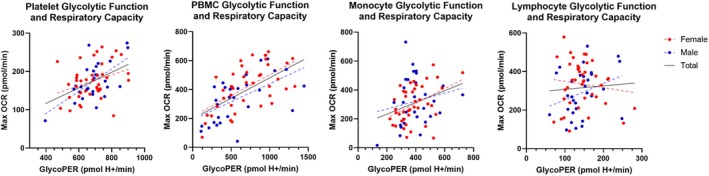
Scatterplots of the association between glycolytic and respiratory capacity. Unadjusted (M0) glycolytic capacity is associated with respiratory capacity in platelets, PBMCs, monocytes, and lymphocytes. GlycoPER, glycolytic proton efflux rate; OCR, oxygen consumption; PBMC, peripheral blood mononuclear cells.

Since Warburg‐like metabolic reprogramming has been linked to aging, we examined the relationship between glycolytic capacity (GlycoPER) and respiratory capacity (Max) in platelets, mixed PBMCs, monocytes, and lymphocytes of men and women (Table 4). We found strong positive correlations between glycolytic capacity and respiratory capacity in platelets (*β* = 0.433, *p* = < 0.001), PBMCs (*β* = 0.566, *p* < 0.001), and monocytes (*β* = 0.337, *p* = 0.005) but not lymphocytes (*β* = 0.026, *p* = 0.832). When stratified by sex, most relationships retained a similar pattern, except for monocytes in which there was no relationship in men (*β* = 0.194, *p* = 0.333). Adjustments for BMI (M1) generally did not significantly affect the relationships, except for platelet Max in women (*β* = 0.272, *p* = 0.108). Scatterplots of each relationship are shown in Figure 2.

**TABLE 4:** Standardized regression parameter estimates for GlycoPER, stratified by sex.

		All		Men		Women	
		GlycoPER		GlycoPER		GlycoPER	
		*β*	*p*	*β*	*p*	*β*	*p*
Platelet max (*n* = 72)	M0	***0.433***	***< 0.001***	***0.558***	***0.003***	***0.331***	***0.039***
	M1	***0.440***	***< 0.001***	***0.579***	***0.002***	0.272	0.108
PBMC max (*n* = 72)	M0	***0.566***	***< 0.001***	***0.596***	***0.001***	***0.540***	***0.001***
	M1	***0.571***	***< 0.001***	***0.590***	***0.002***	***0.558***	***0.001***
Monocyte max (*n* = 71)	M0	***0.337***	***0.005***	0.194	0.333	***0.442***	***0.005***
	M1	***0.324***	***0.008***	0.214	0.312	***0.427***	***0.006***
Lymphocyte max (*n* = 72)	M0	0.026	0.832	0.301	0.104	−0.187	0.258
	M1	0.004	0.975	0.289	0.130	−0.215	0.193

Abbreviations: M0, unadjusted; M1, adjusted for age, BMI.

*Note*: Bold values represent statistical significance (*p* < 0.05).

## 4 Discussion

In this cohort of healthy adults, we found that chronological age was positively associated with measures of bioenergetic capacity in distinct blood cell types. Specifically: age was positively associated with respiration in PBMCs and lymphocytes, with notable sex differences. The associations between age and PBMC respiratory capacity were largely driven by women, with minimal association observed in men. We also observed weak positive correlations between age and platelet bioenergetic capacity in men. Adjustments for BMI generally had weak or no effect on the strength of the associations. Notably, we also observed a strong positive association between glycolytic capacity and respiratory capacity, suggesting collinearity as opposed to age‐related metabolic reprogramming. A key strength of our study lies in its rigorous design, which employed strict inclusion and exclusion criteria aimed at minimizing extrinsic factors—such as comorbidities, medication use, frailty, etc.—thereby centering our study on “normal,” non‐pathological aging. Additionally, our comprehensive bioenergetic profiling approach expands on previous work by including isolated monocytes and lymphocytes, as well as incorporating glycolytic capacity. To our knowledge, this study is the first of its scale and depth to characterize age‐related differences in bioenergetic capacity across multiple blood cell types in healthy adults, offering unique insights into the metabolic underpinnings of healthy aging.

The present study contributes to a more complete understanding of age‐related bioenergetic decline by providing new insights on age‐associated changes in blood cell bioenergetic capacity. We found that respiratory capacity generally increased, rather than decreased, with age in blood cells. This observation aligns with a study by Ehinger et al. (2024) which found that PBMC respiratory capacity increased with age, albeit very slightly, in 308 participants. It is important to note methodological differences compared to our study; they utilized high‐resolution oxygraph respirometry (Oroboros Instruments, Innsbruck, Austria) while we opted for high‐throughput Seahorse‐based respirometry. While maximal respiration on the Seahorse system can be considered akin to electron transfer system maximal capacity (MaxETS) on the Oroboros system, differences in metabolic fuels and uncoupler titration make them technically distinct. Further, Ehinger et al. (2024) included hospital patients with known neurodegenerative movement disorders, patients with sepsis, and pediatric patients in their sample along with healthy controls. Despite these differences, we found similar trends using regression analysis across the lifespan. Other studies investigating age‐related bioenergetic differences have reported conflicting findings. For example, Silaidos et al. (2024) found age‐related decreases in complex‐IV respiration and ATP content in freshly isolated PBMCs of 58 healthy participants. Using cryopreserved PBMCs, Dieter et al. (2025) also reported lower respiration in PBMCs from older adults across multiple bioenergetic parameters. Notably, both these studies employed high‐resolution oxygraph respirometry and treated age as a binary categorical variable (“young” vs. “old”), as opposed to a continuous numerical variable. A different study by Alonso et al. (2021) found an age‐related decrease in multiple PBMC respiration parameters in 38 healthy participants using Seahorse respirometry, although a large proportion of their sample included children and adolescents. In contrast to our PBMC findings, our findings in platelet respiratory capacity interestingly showed minimal age‐related differences consistent with results from Ehinger et al. (2024) and Alonso et al. (2021), though other studies have reported decreases in respiratory capacity with age in platelets (Fišar et al. 2023; Braganza et al. 2019).

Overall, these inconsistencies highlight the importance of considering biological context and methodological rigor in studies of aging. Variation in inclusion and exclusion criteria across studies must be carefully considered when interpreting results. For example, studies such as Fišar et al. (2023) and Braganza et al. (2019) primarily excluded individuals with cognitive impairment or neuropsychiatric disorders, while our criteria expanded to also exclude other age‐related pathological conditions such as diabetes, cardiovascular conditions, and mobility impairments, among others. Similarly, studies such as Ehinger et al. (2024) and Alonso et al. (2021) included pediatric participants, broadening their scope beyond adult aging. As such, our findings are particularly relevant and most applicable to adults on a healthy aging trajectory. It is plausible that circulating cells from individuals who age without pathology or comorbidities, as represented in our cohort, naturally exhibit higher respiratory capacity compared to cells from individuals who age with functional decline or develop age‐related conditions. This distinction underscores the critical role of cohort selection and biological context in interpreting age‐related bioenergetic differences. Another methodological distinction is our use of high‐throughput Seahorse‐based respirometry as opposed to high‐resolution Oroboros‐based respirometry. Both systems have been employed for blood‐based respirometric analysis; we previously highlighted which systems were employed by various studies. We recognize that the differences inherent between both analytical systems may also contribute to conflicting findings.

While many blood‐based bioenergetic studies have focused on mixed PBMCs, our investigation of isolated components provides new insights into cell‐specific influences on overall PBMC bioenergetic capacity. It is evident that unique cell types within a larger population of cells exhibit different degrees of bioenergetic decline with age. For example, although we found a significant association between age and lymphocyte Basal, that pattern was not present in mixed PBMC Basal. A recent study by Gebhardt et al. demonstrated even further distinct bioenergetic differences between lymphocyte populations (CD4, CD8, etc.) (Gebhardt et al. 2025). In our study, although age was significantly associated with mixed PBMC Max, SRC, and GlycoPER in women, those trends were not observed in either isolated monocytes or lymphocytes. Interestingly, this is in contrast with other studies that report decreased maximal respiratory capacity in monocytes from older adults. It is possible that variations in isolation methods contribute to this disparity. For example, a study by Pence and Yarbro included CD16 depletion for isolation of classical monocytes (CD14 + CD16−) without intermediate (CD14 + CD16+) or non‐classical (CD14^dim^CD16+) monocytes, resulting in removal of about 25% of monocytes from the population (Pence and Yarbro 2018). In contrast, our methods do not distinguish between monocyte subtypes. Further, the SD‐NSC featured a more extensive list of health‐based exclusion criteria so our cohort may differ in overall baseline health status.

Notably, we also found clear differences between men and women in the relationship between age and blood cell bioenergetic capacity. It is well established that mitochondrial function differs between men and women. Previous work from our group and others highlights significant differences in the blood bioenergetic profiles between men and women (Mahapatra et al. 2024; Silaidos et al. 2024). Although findings from Mahapatra et al. (2024) suggested that the difference could be due to a difference in PBMC composition between men and women, our numbers suggest that, in this sample, that is not the case. It is also important to note that those findings focused solely on older adults, with an age range of 55–95 years. A pilot study by Silaidos et al. (2024) reported a similar trend of higher PBMC respiration in women compared to men. However, a meta‐analysis by Junker et al. (2022) found wide variability in sex‐specific mitochondrial function of various tissues. However, a closer look at the studies which include blood cells reveals that there were indeed differences between men and women, but they are distinct from the patterns seen in skeletal muscle. For example, platelet respiration was higher in men across all evaluated studies, but skeletal muscle respiration did not significantly differ between men and women across their respective studies. This highlights the importance of biological context in studies of aging. While such granular analyses reduce external validity, our findings provide clear evidence that the differences between different cell types and between men and women can be striking.

While we found little association between glycolytic capacity and age, we found a strong positive association between glycolytic capacity and respiratory capacity across all four cell types. Some studies have recently adapted the Warburg Effect hypothesis to aging cells, positing that age‐related decline in mitochondrial function can lead to an uptick in aerobic glycolysis (Feng et al. 2016). This has been observed in fruit flies, worms, and human primary and cultured cells (Feng et al. 2016; Morris et al. 2020; Traxler et al. 2022). Our results showed that individuals with lower respiratory capacity tend to also have lower glycolytic function, not higher. Because our data were normalized to cell counts, this observation cannot be attributed to lower absolute cell numbers. Nonetheless, our findings indicate that the metabolic shift from oxidative phosphorylation to glycolysis does not occur in blood cells of healthy adults. It is possible that blood cells are more susceptible to metabolic reprogramming following a pathological response, rather than because of healthy aging. In particular, lymphocyte activation induces significant metabolic reprogramming to support bioenergetic demands, including upregulation of glycolysis (Yazicioglu et al. 2024). It may be that non‐pathological aging has little impact on blood cell bioenergetic capacity when metabolic shifts are more tightly linked to the cells' physiological functions.

We intentionally focused on individuals free from comorbidities that are associated with human aging. Previously, work from our group and others utilized blood‐based bioenergetic profiling to examine associations with cognitive impairments associated with MCI and AD as well as functional impairments associated with mobility disability (Tyrrell et al. 2015; Herpich et al. 2021; Huang et al. 2024; Apaijai et al. 2020; Sharma et al. 2021; Mahapatra et al. 2023; Dieter et al. 2025). Such studies have led to a generalized notion that older age itself may be associated with lower mitochondrial respiratory capacity in blood cell populations. This distinction is important, as many previous studies on age‐related bioenergetics focus on individuals with age‐related conditions or impairments rather than healthy participants. However, the findings presented in this study, along with work by Ehinger et al. (2024) challenge this generalization by demonstrating that aging itself does not necessarily entail a universal decline in bioenergetic capacity, but rather is associated with an increase in capacity in certain cell populations. Specifically, our findings showed that age is associated with higher blood cell bioenergetic capacity in healthy, disease‐free adults, which has important implications on our collective understanding of healthy aging and mitochondrial function. While our study does not specifically compare cellular bioenergetic capacity between lower and higher functioning older adults, we posit that the elevated respiratory capacity observed in the older individuals from our cohort may reflect their overall health status and absence of chronic disease. It is likely that older individuals who exhibit lower bioenergetic capacity are more susceptible to age‐related functional decline and frailty, and thus would not be represented by this cohort. A key question arising from this is why cells from healthy older adults exhibit higher bioenergetic capacity compared to cells from younger individuals. Some possible explanations include diminished mitochondrial efficiency with age (Lanza et al. 2005; Yaniv et al. 2013) or inflammaging (López‐Armada et al. 2013). Although we did not directly explore these possibilities, these findings ultimately underscore the importance of considering bioenergetic capacity as a context‐dependent, dynamic marker of healthy aging.

This work is not without limitations. While our careful selection of participants with strict exclusion criteria helps to minimize biological variability, it also leads to a small and relatively homogenous sample of adults (e.g., lower racial diversity, educational attainment, etc.). Experimentally, we did not include inter‐plate controls on each Agilent Seahorse experiment, which may result in some between‐plate variance. We minimized these effects with regular equipment maintenance and bulk preparation of reagents for the entire study. Immunomagnetic selection of immune cells may have consequences on cellular bioenergetics and result in data that does not adequately reflect true basal metabolism. While we opted to separate mixed PBMCs into constituent monocytes and lymphocytes, we did not further differentiate between monocyte or lymphocyte subtypes (i.e., classical vs. non‐classical monocytes or T‐lymphocytes vs. B‐lymphocytes), of which there are known functional differences. Additionally, we did not investigate mitochondrial content in any cells, which may or may not have influenced the associations observed in our bioenergetic outcomes. However, we normalized our bioenergetic outcomes to cell number, so observations are still directly comparable at the cellular level. Similarly, we did not measure hormone levels or menopausal status, which may potentially confound our female data.

In conclusion, in this sample of healthy adults across the human life course, age was positively associated with bioenergetic capacity in PBMCs and lymphocytes. Platelets exhibit a similar trend, but only in men, while PBMCs exhibit their positive association more strongly in women. These findings are seemingly in contrast with other studies that report an age‐related decrease in cellular bioenergetic capacity. However, our study is unique based on our strict selection criteria, additional isolated blood cell types, and sex‐stratified analysis. While we recognize that both monocyte and lymphocyte populations can be further subdivided (e.g., T and B lymphocytes, CD4 vs. CD8 T lymphocytes, etc.), our inclusion of isolated lymphocytes and monocytes is a step forward from other studies which focus solely on mixed PBMC populations in healthy adults with no further subdivision. The results of our study are applicable to healthy, disease‐free adults, with our finding of elevated bioenergetic capacity in older adults requiring further investigation for causation. Nonetheless, in the context of literature demonstrating diminished age‐related bioenergetic capacity, our results challenge the notion of a universal “age‐related bioenergetic decline,” emphasizing instead the importance of biological context—including cell type, sex, and health status—in interpreting mitochondrial changes with age. These findings advance our understanding of age‐related bioenergetic decline and underscore the potential of blood‐based bioenergetic profiling as a valuable tool for elucidating the mechanisms of biological aging.

## Author Contributions

Conceptualization: Howard J. Phang, Stephanie R. Heimler, David Wing, Anthony J.A. Molina. Methodology: Howard J. Phang, Jaclyn Bergstrom, Benjamin Keri, Stephanie R. Heimler, Stephen Dozier, Lina M. Scandalis, David Wing, Daniel Moreno, Nina N. Sun. Investigation: Howard J. Phang, Benjamin Keri, Stephanie R. Heimler, Stephen Dozier, David Wing, Daniel Moreno, Nina N. Sun. Supervision: Lina M. Scandalis, David Wing, Anthony J.A. Molina. Writing – original draft: Howard J. Phang, Jaclyn Bergstrom. Writing – review and editing: Jaclyn Bergstrom, Benjamin Keri, Stephanie R. Heimler, Stephen Dozier, Lina M. Scandalis, David Wing, Daniel Moreno, Nina N. Sun, Anthony J.A. Molina.

## Funding

This work was supported by the National Institutes of Health [PI, Gerald Shadel; Grant, P30AG068635].

## Conflicts of Interest

The authors declare no conflicts of interest.

## Supporting information


**Data S1:** Table S1: Sex stratified participant characteristics.
**Table S2:** Standardized regression parameters of age for blood cell bioenergetic parameters—subset with physical activity.
**Table S3:** Standardized regression parameters of age for blood cell bioenergetic parameters, stratified by sex—subset with physical activity.

## Data Availability

Data will be made available upon reasonable request to the authors.
