# Supplementary material for: Blood Cell Mitochondrial Respiration Increases With Age and Varies by Sex in Healthy Adults
Source: Aging Cell. 2026 Jan 21;25(2):e70387. doi: 10.1111/acel.70387 (PMC12823460; doi:10.1111/acel.70387)
Supplement: Supplementary file 1 — Data S1: Table S1: Sex stratified participant characteristics. Table S2: Standardized regression parameters of age for blood cell bioenergetic parameters—subset with physical activity. Table S3: Standardized regression parameters of age for blood cell bioenergetic parameters, stratified by sex—subset with physical activity. [file ACEL-25-e70387-s001.docx]

**eTable 1: Sex stratified participant characteristics**

| ***Men*** |  |  |  |  |  |  |
| --- | --- | --- | --- | --- | --- | --- |
|  | ***N*** | ***All*** | ***20-40*** | ***40-60*** | ***60-80+*** | ***p-value*** |
| ***N*** |  | *31* | *10* | *7* | *14* |  |
| ***Body max index (BMI), mean (SD)*** | 31 | 24.65 (2.75) | 25.13 (2.95) | 24.50 (3.26) | 24.37 (2.49) | 0.800 |
| ***Platelet count (million/mL), mean (SD)*** | 31 | 170.01 (34.38) | 176.08 (28.35) | 154.44 (32.56) | 173.46 (38.86) | 0.402 |
| ***PBMC count (million/mL), mean (SD)*** | 31 | 1.60 (0.45) | 1.75 (0.49) | 1.48 (0.33) | 1.55 (0.46) | 0.408 |
| ***Monocyte count (million/mL), mean (SD)*** | 30 | 0.34 (0.08) | 0.38 (0.10) | 0.31 (0.04) | 0.33 (0.08) | 0.203 |
| ***Lymphocyte count (million/mL), mean (SD)*** | 31 | 1.26 (0.39) | 1.37 (0.43) | 1.16 (0.31) | 1.24 (0.40) | 0.528 |
| ***Monocyte % of PBMCs, mean (SD)*** | 31 | 24.51 (3.94) | 25.57 (3.74) | 24.48 (3.25) | 23.77 (4.45) | 0.561 |
| ***Lymphocyte % of PBMCs, mean (SD)*** | 31 | 74.58 (4.05) | 73.72 (3.76) | 74.32 (2.89) | 75.33 (4.80) | 0.634 |
| ***Women*** |  |  |  |  |  |  |
|  | ***N*** | ***All*** | ***20-40*** | ***40-60*** | ***60-80+*** | ***p-value*** |
| ***N*** |  | *41* | *10* | *13* | *18* |  |
| ***Body max index (BMI), mean (SD)*** | 41 | 23.89 (3.01) | 24.74 (3.19) | 24.85 (2.42) | 22.73 (3.05) | 0.089 |
| ***Platelet count (million/mL), mean (SD)*** | 41 | 203.71 (48.25) | 208.02 (77.33) | 199.88 (33.82) | 204.07 (38.51) | 0.925 |
| ***PBMC count (million/mL), mean (SD)*** | 41 | 1.64 (0.52) | 2.07 (0.63) | 1.49 (0.24) | 1.51 (0.50) | 0.008 |
| ***Monocyte count (million/mL), mean (SD)*** | 41 | 0.32 (0.10) | 0.36 (0.13) | 0.29 (0.05) | 0.33 (0.09) | 0.196 |
| ***Lymphocyte count (million/mL), mean (SD)*** | 41 | 1.32 (0.46) | 1.71 (0.53) | 1.20 (0.24) | 1.19 (0.44) | ***0.006*** |
| ***Monocyte % of PBMCs, mean (SD)*** | 41 | 23.52 (5.09) | 20.22 (4.45) | 22.75 (3.79) | 25.91 (5.25) | ***0.011*** |
| ***Lymphocyte % of PBMCs, mean (SD)*** | 41 | 74.95 (5.72) | 77.09 (7.24) | 76.22 (3.92) | 72.85 (5.46) | 0.106 |

*Notes: SD, standard deviation; PBMC, peripheral blood mononuclear cells*

**eTable 2: Standardized regression parameters of age for blood cell bioenergetic parameters – subset with physical activity**

|  |  | ***Basal*** | | ***Max*** | | ***SRC*** | | ***GlycoPER*** | |
| --- | --- | --- | --- | --- | --- | --- | --- | --- | --- |
|  |  | ***β*** | ***p-value*** | ***β*** | ***p-value*** | ***β*** | ***p-value*** | ***β*** | ***p-value*** |
| ***Platelet*** | ***M0*** | 0.102 | 0.135 | 0.109 | 0.133 | 0.078 | 0.280 | ***0.170*** | ***0.011*** |
|  | ***M1*** | 0.095 | 0.178 | 0.100 | 0.181 | 0.068 | 0.367 | ***0.147*** | ***0.030*** |
|  | ***M2*** | 0.096 | 0.184 | 0.100 | 0.191 | 0.068 | 0.377 | ***0.149*** | ***0.032*** |
| ***PBMCs*** | ***M0*** | 0.081 | 0.258 | ***0.150*** | ***0.036*** | ***0.157*** | ***0.028*** | 0.135 | 0.055 |
|  | ***M1*** | 0.048 | 0.510 | 0.129 | 0.080 | 0.145 | 0.052 | 0.108 | 0.126 |
|  | ***M2*** | 0.026 | 0.721 | 0.104 | 0.147 | 0.123 | 0.093 | 0.09 | 0.203 |
| ***Monocyte*** | ***M0*** | 0.039 | 0.575 | -0.010 | 0.886 | -0.018 | 0.797 | 0.071 | 0.308 |
|  | ***M1*** | 0.057 | 0.419 | 0.018 | 0.801 | 0.009 | 0.898 | 0.086 | 0.231 |
|  | ***M2*** | 0.071 | 0.324 | 0.007 | 0.922 | -0.005 | 0.941 | 0.095 | 0.193 |
| ***Lymphocyte*** | ***M0*** | ***0.235*** | ***0.001*** | ***0.142*** | ***0.045*** | 0.121 | 0.090 | 0.095 | 0.192 |
|  | ***M1*** | ***0.262*** | ***<0.001*** | ***0.174*** | ***0.016*** | ***0.152*** | ***0.038*** | 0.110 | 0.141 |
|  | ***M2*** | ***0.261*** | ***<0.001*** | ***0.161*** | ***0.028*** | 0.138 | 0.062 | 0.124 | 0.101 |

*Notes: M0 = unadjusted; M1: adjusted for BMI; M2: adjusted for BMI and physical activity; SRC, spare respiratory capacity; GlycoPER, glycolytic proton efflux rate; PBMC, peripheral blood mononuclear cells*

**eTable 3: Standardized regression parameters of age for blood cell bioenergetic parameters, stratified by sex – subset with physical activity**

|  |  | ***Basal*** | | ***Max*** | | ***SRC*** | | ***GlycoPER*** | |
| --- | --- | --- | --- | --- | --- | --- | --- | --- | --- |
|  |  | ***β*** | ***p-value*** | ***β*** | ***p-value*** | ***β*** | ***p-value*** | ***β*** | ***p-value*** |
| ***Men N=29*** | |  |  |  |  |  |  |  |  |
| ***Platelet*** | ***M0*** | 0.189 | 0.090 | ***0.221*** | ***0.040*** | 0.180 | 0.093 | 0.202 | 0.075 |
|  | ***M1*** | 0.199 | 0.078 | ***0.231*** | ***0.035*** | 0.187 | 0.087 | 0.200 | 0.083 |
|  | ***M2*** | ***0.230*** | ***0.049*** | ***0.250*** | ***0.029*** | 0.193 | 0.092 | 0.217 | 0.069 |
| ***PBMC*** | ***M0*** | -0.082 | 0.460 | 0.047 | 0.673 | 0.098 | 0.387 | -0.027 | 0.807 |
|  | ***M1*** | -0.087 | 0.451 | 0.031 | 0.786 | 0.079 | 0.489 | -0.028 | 0.805 |
|  | ***M2*** | -0.126 | 0.250 | 0.010 | 0.932 | 0.071 | 0.548 | -0.066 | 0.537 |
| ***Monocyte*** | ***M0*** | -0.123 | 0.238 | -0.047 | 0.661 | -0.028 | 0.798 | -0.049 | 0.645 |
|  | ***M1*** | -0.111 | 0.288 | -0.043 | 0.697 | -0.025 | 0.819 | -0.035 | 0.732 |
|  | ***M2*** | -0.090 | 0.405 | -0.039 | 0.738 | -0.024 | 0.835 | -0.018 | 0.863 |
| ***Lymphocyte*** | ***M0*** | ***0.250*** | ***0.017*** | 0.145 | 0.182 | 0.124 | 0.256 | 0.025 | 0.821 |
|  | ***M1*** | ***0.259*** | ***0.016*** | 0.156 | 0.159 | 0.134 | 0.228 | 0.030 | 0.790 |
|  | ***M2*** | ***0.234*** | ***0.031*** | 0.158 | 0.173 | 0.139 | 0.232 | 0.025 | 0.833 |
|  |  |  |  |  |  |  |  |  |  |
| ***Women N=37*** | |  |  |  |  |  |  |  |  |
| ***Platelet*** | ***M0*** | 0.037 | 0.674 | 0.022 | 0.826 | -0.002 | 0.987 | 0.154 | 0.071 |
|  | ***M1*** | -0.015 | 0.874 | -0.031 | 0.769 | -0.052 | 0.624 | 0.101 | 0.242 |
|  | ***M2*** | -0.024 | 0.790 | -0.039 | 0.714 | -0.054 | 0.615 | 0.094 | 0.285 |
| ***PBMC*** | ***M0*** | 0.204 | 0.035 | ***0.231*** | ***0.017*** | ***0.208*** | ***0.033*** | ***0.263*** | ***0.005*** |
|  | ***M1*** | 0.146 | 0.134 | ***0.215*** | ***0.036*** | ***0.211*** | ***0.043*** | ***0.212*** | ***0.025*** |
|  | ***M2*** | 0.131 | 0.178 | 0.188 | 0.054 | 0.184 | 0.065 | ***0.205*** | ***0.033*** |
| ***Monocyte*** | ***M0*** | 0.174 | 0.062 | 0.022 | 0.811 | -0.008 | 0.929 | 0.162 | 0.092 |
|  | ***M1*** | ***0.205*** | ***0.040*** | 0.084 | 0.385 | 0.054 | 0.578 | 0.167 | 0.106 |
|  | ***M2*** | ***0.212*** | ***0.038*** | 0.068 | 0.482 | 0.035 | 0.716 | 0.170 | 0.108 |
| ***Lymphocyte*** | ***M0*** | ***0.230*** | ***0.016*** | 0.145 | 0.137 | 0.123 | 0.210 | 0.152 | 0.128 |
|  | ***M1*** | ***0.276*** | ***0.006*** | ***0.201*** | ***0.050*** | 0.176 | 0.089 | 0.176 | 0.100 |
|  | ***M2*** | ***0.285*** | ***0.005*** | 0.181 | 0.073 | 0.154 | 0.129 | 0.198 | 0.059 |

*Notes: M0 = unadjusted; M1: adjusted for BMI; M2: adjusted for BMI and physical activity; SRC, spare respiratory capacity; GlycoPER, glycolytic proton efflux rate; PBMC, peripheral blood mononuclear cells*
